# What is known about the effects of exercise or training to reduce skeletal muscle impairments of patients with myotonic dystrophy type 1? A scoping review

**DOI:** 10.1186/s12891-019-2458-7

**Published:** 2019-03-05

**Authors:** Marie-Pier Roussel, Marika Morin, Cynthia Gagnon, Elise Duchesne

**Affiliations:** 1 Département des sciences de la santé, physiothérapie, Université du Québec à Chicoutimi, 555, boulevard de l’Université, Chicoutimi, Quebec G7H 2B1 Canada; 2 Groupe de recherche interdisciplinaire sur les maladies neuromusculaires, Centre intégré universitaire de santé et de services sociaux du Saguenay–Lac-St-Jean, 2230 rue de l’Hôpital, Saguenay, Québec Canada; 3 Centre de recherche Charles-Le Moyne – Saguenay–Lac-Saint-Jean sur les innovations en santé, 2230 rue de l’Hôpital, Saguenay, Québec, Canada., Longueuil, Québec Canada; 4 Faculté de médecine et des sciences de la santé, Université de Sherbrooke, 3001, 12e Avenue Nord, Sherbrooke, Québec Canada

**Keywords:** Myotonic dystrophy type 1, Exercise, Training program, Rehabilitation interventions, Muscle weakness, Muscle atrophy

## Abstract

**Background:**

Myotonic dystrophy type 1 (DM1) is a neuromuscular disease characterized by multisystemic involvements including a progressive loss of maximal muscle strength and muscle wasting. Poor lower-limb strength is an important factor explaining disrupted social participation of affected individuals. This review aims to map what is known about the effects of exercise and training programs undertaken to counteract skeletal muscle impairments in DM1 patients.

**Methods:**

Medline, CINAHL and EMBASE databases were searched. Regarding study eligibility, title and abstract of 704 studies followed by 45 full articles were reviewed according to the following eligibility criteria. Inclusion: (1) humans with DM1 and (2) experimental protocol relying on exercise or training. Exclusion: (1) studies that do not evaluate skeletal muscle responses or adaptations, (2) reviews covering articles already included and (3) pharmacological intervention at the same time of exercise or training program.

**Results:**

Twenty-one papers were selected for in-depth analysis. Different exercise or training protocols were found including: acute exercise, neuromuscular electric stimulation, strength training, aerobic training, balance training and multiple rehabilitation interventions. Seven studies reported clinical measurements only, five physiological parameters only and nine both types.

**Conclusion:**

This scoping review offers a complete summary of the current scientific literature on the effect of exercise and training in DM1 and a framework for future studies based on the concomitant evaluation of the several outcomes in present literature. Although there were a good number of studies focusing on clinical measurements, heterogeneity between studies does not allow to identify what are the adequate training parameters to obtain exercise or training-induced positive impacts on muscle function. Scientific literature is even more scarce regarding physiological parameters, where much more research is needed to understand the underlying mechanisms of exercise response in DM1.

**Electronic supplementary material:**

The online version of this article (10.1186/s12891-019-2458-7) contains supplementary material, which is available to authorized users.

## Background

Myotonic dystrophy type 1 (DM1) is an autosomal dominant degenerative neuromuscular disease [1–3] caused by a nucleotide triplet (CTG) repeat expansion within the 3′ untranslated region of the *Dystrophy Myotonic Protein Kinase* (*DMPK*) gene [4]. The discovery of the defective gene was made in 1992, which subsequently allowed a more accurate diagnosis [4]. CTG repeat length is usually moderately correlated with earlier disease onset and more severe symptoms [5]. There are four to five recognized phenotypes: congenital, infantile/childhood, juvenile, classical/adult and late-onset [5].

DM1 is a multisystemic disease that affects skeletal muscles and cardiac, digestive, nervous and endocrine systems, to name a few. Typical signs and symptoms include myotonia, heart arrhythmias, cataracts, daytime sleepiness, cognitive impairments, apathy and muscle wasting and weakness. The latter is a major concern for affected people, which can lose up to 30.3 to 54.5% of maximum strength on a 9 year period with a progression pattern from distal to proximal muscles [6]. A recent study has shown that even DM1 patients classified in the first two grades of Muscle Impairment Rating Scale (MIRS) [7], which are considered to have no limb muscles weakness, show a 11.3 to 24.1% of maximal strength loss in lower limb muscle groups compared to predicted values [8]. Muscle weakness is a major culprit for restrictions in activities of daily living and social roles [9].

Although the physiological mechanisms are not yet fully understood, one of the main mechanisms linking the CTG repetitions to the symptoms in DM1 is the accumulation of toxic CUG RNA causing splicing difficulties by sequestrating RNA-binding proteins such as muscle blindlike-1 (MBNL-1) [10]. Originating from this, many physiological impairments have been identified in skeletal muscle affected by DM1 that could contribute to muscle weakness. At a histological level, preferential atrophy of type I myofibers, higher proportion of centrally nucleated fibers, fibrosis and fat infiltration have been reported [11]. Moreover, level of expression of glycogen synthase kinase 3 beta (GSK3β), a negative regulator of muscle protein synthesis, is increased [12]. Thereafter, myogenic capacity is also perturbed as demonstrated by decreased proliferative capacity and premature senescence of satellite cells (muscle stem cells) and delayed fusion and differentiation of myoblasts (activated satellite cells) [13–17]. An increase in protein degradation and a decrease in protein synthesis [1, 18–20], as well as perturbed local and systemic inflammatory statuses [21, 22], have also been reported. Finally, a mouse model study has also shown that calcineurin is overexpressed in DM1, arguing that it may be a compensatory mechanism due to its essential role in muscle hypertrophy [23]. This study also showed that symptoms worsened in affected mice where calcineurin was downregulated [23]. These findings only present an incomplete view of muscle physiology in DM1, and knowledge about immediate responses and long-term adaptations to exercise or training are essential for a complete understanding.

One of the main strategies to counter maximal muscle strength loss and muscle wasting is through exercise and training. However, to be effective, exercise and training need to be specifically adapted to a population and based on an appropriate dosage. Furthermore, a rationale based on physiological knowledge is a key point to properly adapt training to populations with a specific pathology. In DM1 population, while the underlying mechanism remain unclear given the small number of studies along with the small number subjects included in these, training-induced positive skeletal muscle adaptation seems possible in DM1. Indeed, it has been shown that training can induce increase in myofiber cross-sectional area without causing any histopathological abnormalities [3, 24]. These results suggest that, in addition to the positive clinical impact of exercise in DM1 such as muscle strength gains, exercise should physiologically work in this disease. A systematic review has shown strength training to be safe [25], but has not concluded about its effect on skeletal muscle impairments given the insufficient body of literature. However, the Cochrane review did not look in detail at the type of training nor at the type of intervention outcomes (fundamental or clinical measures) that were studied. This last aspect is particularly important because skeletal muscle responses and adaptations to exercise and training do not limit themselves to maximal strength or endurance gains; many changes can occur at a physiological, functional or participation levels. A better understanding of the physiological responses and adaptations can also help uncover new intervention possibilities, such as a pharmaceutical agent combined with exercise or training to limit muscle impairments. Therefore, the main purpose of this scoping review is to map the existing literature relative to the effects of exercise and training undertaken to limit skeletal muscle impairments in DM1 and thereby identify gaps in the literature and informing where more research is needed. Cochrane systematic review methodology, which is more restrictive in their study selection, cannot be used to undertake this type of mapping of the literature.

## Methods

In this review, the framework Methodology for JBI Scoping Reviews was used to develop the methods [26]. This framework is the best to use in this review because it has been designed to be broad enough to include any kind of existing scientific literature, thus allowing the most complete mapping on the desired subject and allowing to better achieve the objective of this study.

### Research question

The scoping review aimed to answer the following question: “What is known about the effects of exercise and training that aim to reduce skeletal muscle impairments of patients with myotonic dystrophy type 1?” The question was built using the PCC (population, context and concept) model [26] where DM1 was the population, exercise or training were the context and muscle responses or adaptations (clinical or physiological) were the concept.

### Identifying relevant studies

A systematic literature search was undertaken on February 16th, 2017 with no date limitations to identify all relevant studies. The search was organised into three main concepts and sub-concepts: concept 1-myotonic dystrophy, concept 2-clinical interventions (2a: exercise training, 2b: rehabilitation), and concept 3-muscle (see Supplemental digital content 1).

These three concepts allow the identification of appropriate keywords thesaurus and synonyms according to each database. Four databases were searched: Additional file 1: Medline (EBSCO), Pubmed, CINAHL (EBSCO) and EMBASE. Duplicates and articles that were not in French or in English were removed (119 articles were removed due to language). The search strategy was reviewed by a knowledge broker. A total of 704 articles were obtained (690 English and 14 French).

### Study selection

Two independent reviewers (M-PR and MM) screened the article titles and abstracts according to the following inclusion and exclusion criteria. Inclusion: (1) humans with DM1 and (2) experimental protocol relying on exercise or training. Exclusion: (1) studies that do not evaluate skeletal muscle responses or adaptations (clinical or physiological), (2) reviews that covered articles that were already included in this scoping review and (3) pharmacological intervention at the same time of the exercise or training protocol. After the initial screening, the remaining articles were fully read and screened according to the same criteria by the same independent reviewers (M-PR and MM). In case of a disagreement, both reviewers discussed about the eligibility of the study. If a consensus was not reached, a third reviewer (ED) was consulted.

### Charting the data

The data from the selected studies were then charted by one reviewer (M-PR) and reviewed by a second reviewer, a second-year physical therapy student with two years of experience as a research trainee in our lab, according to a data extraction grid to ensure standardized data extraction (Table 1). By working exclusively on projects focusing on DM1, both evaluators have some knowledge of the disease. Moreover, their training in physical therapy along with their experience as trainee in wet lab make them familiar with the continuum of outcome measures found in the literature. A beta version of the extraction grid was tested on three articles before the final grid was produced.

**Table 1:** Data extraction grid

Title/Journal	
Author(s)	
Year	
Study eligibility	
Country	
Aims/Objectives	
Diagnostic criteria, inclusion/exclusion criteria and phenotype	
Number of DM1 participants and protocol compliance	
Sex	
Age	
Other clinical manifestations of DM1 considered	
Study design and exercise or training protocol (detailed)	
Muscles targeted by the exercise or training protocol	
Clinical measurements and detailed protocol	
Physiological parameters evaluated	
*P* value, confidence interval	
Results (detailed)	

### Collating, summarizing and reporting the results

The results were summarized under two main themes: outcome measures and exercise or training protocols. The outcome measures were subdivided in clinical measurements and physiological variables (top of Additional file 2). Clinical measurements were categorized as intervention outcomes (patient-reported outcome measures, functional tests, muscle endurance tests, aerobic capacity tests, muscle strength tests and other) and sub-categorized into specific measure types. Physiological variables were categorized as laboratory tool (myography, magnetic resonance imagery (MRI), muscle biopsy analysis and blood tests) and sub-categorized into studied physiological variables. Each of these sub-categories can be seen in Additional file 2. There were two types of exercise or training protocols: single session exercise and training programs. The type of training programs used (far left column of Additional file 2) were categorized as: neuromuscular electric stimulation (NMES), strength training, aerobic training, balance training and multiple rehabilitation interventions. Also, as the detailed protocol of some clinical measurements were not standardized, mostly concerning muscle endurance, aerobic capacity and strength testing, information needed to reproduce them were assessed and classified by two independent reviewers (M-PR and the second-year physical therapy student). If there was a disagreement, a third reviewer would decide (ED).

## Results

**Fig. 1:**
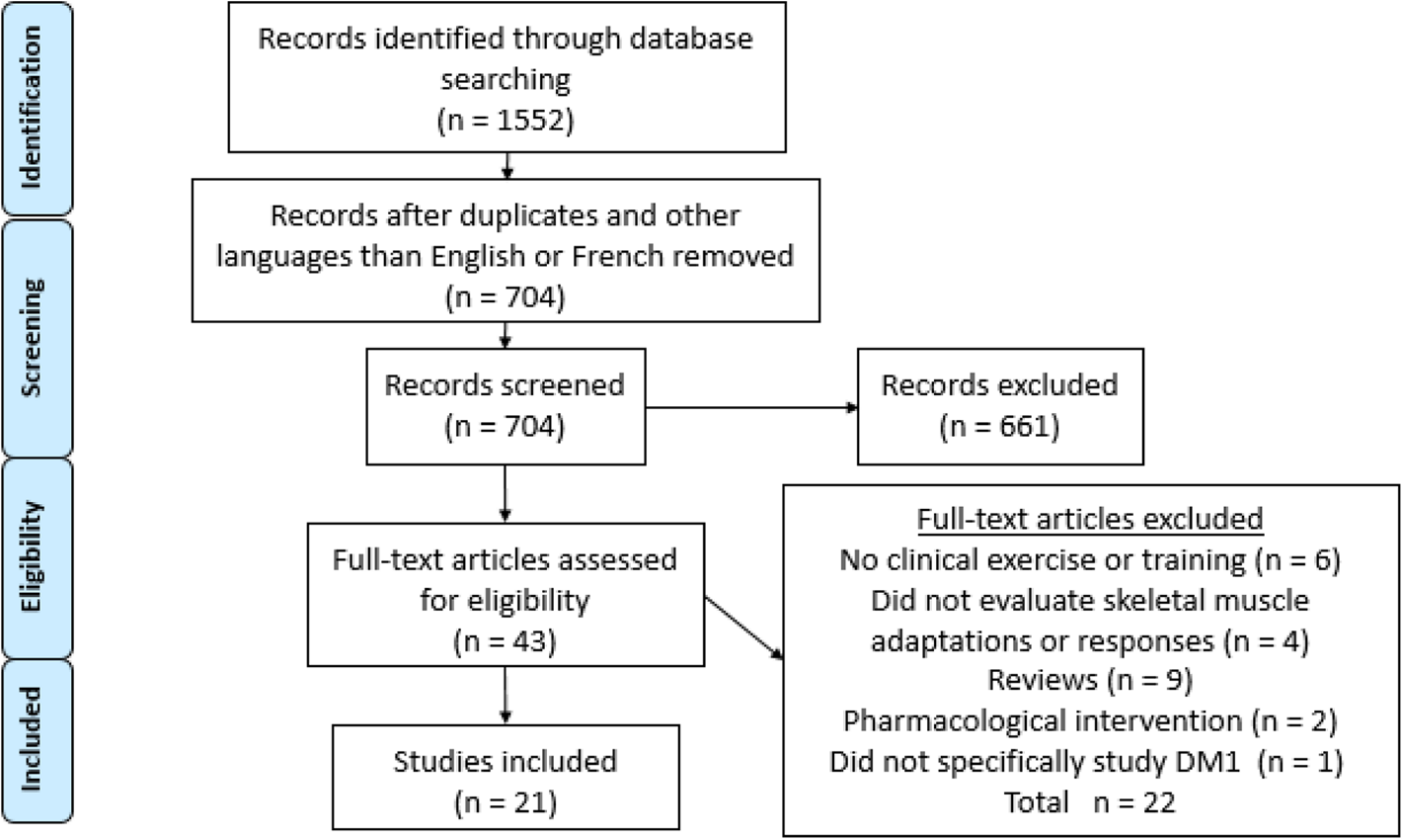
Flow chart results of the systematic literature search according to the PRISMA statement [27]

The detailed steps of the systematic literature search can be found in the PRISMA flow chart (Fig. 1) [27]. A total of 704 papers were identified following the systematic search, from which 43 were selected based on title and abstract. These 43 papers were entirely read and both reviewers (M-PR and MM) excluded 22 papers according to inclusion and exclusion criteria (Fig. 1 for reasons of exclusion).

### Study characteristics

Detailed information regarding the selected papers can be found in Table 2. Within the twenty-one selected studies, four were randomised controlled trials (RCT) [2, 28–30], twelve had before-after designs [3, 24, 31–40], two were cross over studies [41, 42], one was retrospective [43], one was a case study [44] and one was a case report [45]. Sample size varied between 1 and 35 participants depending on the design with most studies with before-after design or RCT with a sample size ranging from 10 to 35 DM1 participants.

**Table 2:** Study Characteristics

Author	Year Country	Title	Design n	Genetic testing Phenotype	Aims	Intervention	Main outcomes
Clinical measurements							
Aldehag et al. [28]	2013 Sweden	Effects of hand-training in persons with myotonic dystrophy type 1 – a randomised controlled cross-over pilot study	RCT 35	Yes Childhood, adult and late onset	To investigate the effects of a hand-training program on grip, pinch and wrist force, manual dexterity and activities of daily living, in adults with DM1.	Mix of supervised and unsupervised 12-week hand strength training with putty.	Improved strength in isometric wrist flexors and in perceived function. Other muscle groups showed a trend towards strength gains. No improved dexterity noted.
Aldehag et al. [41]	2005 Sweden	Effects of a hand training programme in five patients with myotonic dystrophy type 1.	Cross over 5	Yes Adult onset	To evaluate hand function in five patients with DM1 after a 12-week strength training program.	Mix of supervised and unsupervised 12-week hand strength training with putty.	Improved strength in individual muscle testing but not prehension. Improved dexterity. No change in myotonia.
Conraads et al. [45]	2002 Belgium	Importance of physical rehabilitation before and after cardiac transplantation in a patient with myotonic dystrophy: a case report	Case report 1	Not specified Not specified	Case report of the effect of rehabilitation on a patient that has undergone heart transplantation with DM1.	Complete supervised rehabilitation (over several years, with complications).	Improved respiratory function, strength, aerobic endurance and tolerated work load.
Hammarén et al. [35]	2015 Sweden	Effects of a balance exercise programme in myotonic dystrophy type 1: A pilot study	Before after 11	Yes Not specified	To evaluate the effects of balance exercises in adults with DM1 directly after intervention and at follow-up after 12 weeks.	10-week supervised balance training program.	Results reported for every patient individually. Most show quadriceps strength improvement others deterioration, all show ankle dorsiflexor strength deterioration. Most showed improvement in the step test. Varied results in TUG. Diminished speed in 10mwt in most patients.
Kierkegaard et al. [29]	2011 Sweden	Feasibility and effects of a physical exercise programme in adults with myotonic dystrophy type 1: a randomized controlled pilot study	RCT 35	Yes Not specified	To investigate the feasibility and effects of a physical exercise programme on functioning and health-related quality of life in adults with DM1.	14-week supervised varied training program (strength, aerobic, balance, flexibility).	Their exercise program is feasible. No significant results, trends indicate an improvement in 6mwt, timed stands test and TUG times. No adverse effects noticed.
Missaoui et al. [43]	2010 France	Posture and gait abilities in patients with myotonic dystrophy (Steinert disease). Evaluation on the short-term of a rehabilitation program	Retrospective 20	Not specified Not specified	To evaluate the effects of a rehabilitation program in terms of balance, gait and muscle strength in a population of patients with DM1.	Complete supervised rehabilitation.	Significant improvements in strength, BBS, functional reach test and TUG.
Sjögreen et al. [42]	2010 Sweden	The effect of lip strengthening exercises in children and adolescents with myotonic dystrophy type 1.	Cross-over 8	Not specified Congenital and childhood onset	To investigate if regular training with an oral screen could strengthen the lip muscles in children and adolescents with DM1.	16-week home based lip strength training.	Results reported for every patient individually, some show improvements in strength and other have no significant changes. No adverse effects were noted.
Physiological variables							
Barnes et al. [31]	1997 United Kingdom	Skeletal muscle metabolism in myotonic dystrophy A 31P magnetic resonance spectroscopy study	Before after 31	Yes Different severities^a^	Evaluate muscle bioenergetics in DM1 patients at rest and during exercise.	Aerobic and ischemic acute exercise in hands and triceps.	More rapid depletion of PCr and increased ATP utilisation indicates an increased metabolic demand to exercise. Diminished acidification post exercise in DM1 patients. Suggestion of mitochondrial and glycogenolysis defect.
Rehunen et al. [37]	1985 Finnland	High-energy phosphate compounds in slow-twitch and fast-twitch muscle fibres. Changes during exercise in some neuromuscular diseases	Before after 5	N/A Not specified	To ascertain whether patients with neuromuscular diseases have any differences in the level and use of the high-energy phosphates, ATP and creatine phosphate, in ST and FT muscle fibres.	Acute 30s ergocycle exercise.	Higher but non-significant depletion in PCr and ATP post exercise in DM1 vs controls. Higher but non-significant levels of PCr at rest in DM1 vs controls.
Siciliano et al. [38]	2001 Italy	Coenzyme Q10, exercise lactate and CTG trinucleotide expansion in myotonic dystrophy	Before after 35	Yes Not specified	To evaluate, in DM1 patients, CoQ10 blood levels and relate them to the degree of CTG expansion as well as to the amount of lactate production in exercising muscle as indicator of mitochondrial dysfunction.	Acute repeated 3 min bouts of exercise with 2 min rest until unable to complete a 3 min bout on ergocycle.	Increased lactate levels during exercise and at rest in DM1 vs controls. Anaerobic threshold took place earlier in DM1. Lactate recovery similar to controls. Inverse correlation between CoQ10 levels and exercise lactate levels suggesting mitochondrial defects in DM1.
Taylor et al. [39]	1993 United Kingdom	Skeletal muscle bioenergetics in myotonic dystrophy	Before after 10	No Not specified	To determine the skeletal muscle bioenergetics in DM1.	Four maximal repetitions of acute finger flexion exercises.	Abnormalities in intracellular inorganic phosphate and proton accumulation during exercise. Decreased intracellular acidification during exercise. No evidence of impaired mitochondrial or glycogenolytic function.
Trenell et al. [44]	2006 Australia	Exercise and myotonic dystrophy: a 31P magnetic resonance spectroscopy and magnetic resonance imaging case study	Case study 1	Yes Not specified	To determine the effect of a 12-week aerobic training program on muscle metabolites in a patient with DM1.	12-week aerobic supervised training on ergocycle.	Normalisation of metabolites post training, unchanged pH and moderate increase in muscle volume.
Both clinical measurements and physiological variables							
Chisari et al. [32]	2013 Italy	Chronic muscle stimulation improves muscle function and reverts the abnormal surface EMG pattern in myotonic dystrophy: a pilot study	Before after 5	Yes Not specified	To evaluate the effects of chronic electrical stimulation both on functional and electrical properties of muscle in DM1 patients.	Home-based NMES twice a day for fifteen or thirty days on the *Tibialis anterior* muscle.	Improved strength in some patients, improved 10mwt time in all patients, normalisation of SMEG pattern.
Cudia et al. [33]	2016 Italy	Effects of Functional Electrical Stimulation Lower Extremity Training in Myotonic Dystrophy Type I	Before after with control group 8	Yes Not specified	Evaluate the safety and effectiveness of functional electrical stimulation lower extremity training in DM1.	Supervised NMES while cycling or standard strengthening exercise program for controls.	Improved strength and 6mwt distance in both groups with greater improvement in the functional electrical stimulation group. No adverse effects of functional electrical stimulation.
Esposito et al. [34]	2017 Italy	Electromechanical delays during a fatiguing exercise and recovery in patients with myotonic dystrophy type 1	Before after with control group 14	Yes Age of onset: 15 ± 18 years^a^	Evaluate the changes in the different delays components during a fatiguing exercise in patients with DM1.	Rhythmic isometric contractions of quadriceps and *tibialis anterior* at 50% maximal strength until exhaustion.	No difference in time until exhaustion between participants with DM1 and matched controls. Delays components were longer in DM1 compared to controls, especially in distal muscles. Delays components recovery was slower in DM1 compared to controls.
Lindeman et al. [30]	1995 Netherlands	Strength training in patients with myotonic dystrophy and hereditary motor and sensory neuropathy: a randomized clinical trial	RCT 30	No Two congenital and 28 adult onset	To determine whether short-term muscle strength training is efficacious for improving impairments, disabilities and handicap in patients with DM1.	24-week home based strength training with weights.	No significant changes in strength, the weakest participants show no change while a trend in the other participants shows small gains. No adverse effects noted. Trend shows an increase in endurance. Some qualitative items show a significant improvement. Membrane permeability did not change.
Lindeman et al. [2]	1999 Netherlands	Progressive resistance training in neuromuscular patients. Effects on force and surface EMG	RCT 33	No Two congenital and 31 adult onset	To determine the effect of strength training on surface electromyography and muscle strength in patients with DM1 and other neuromuscular diseases.	24-week home based strength training with weights.	No significant changes but trends show an improvement of the quadriceps. No significant changes in SEMG. There was a significant improvement in endurance.
Nitz et al. [36]	1999 Australia	A study of repeated lateral pinch grip in myotonic dystrophy	Before after 10	Yes Not specified	To investigate the response of hand and forearm muscles during repeated lateral pinch grip efforts in DM1 compared to controls.	10 acute lateral pinch grip exercises.	In DM1 participants, diminished strength, slower grip rate development and release vs controls. Increased electrical activity in DM1 vs a diminution in controls. Fatigue responses similar in both groups.
Orngreen et al. [3]	2005 Denmark	Aerobic training in patients with myotonic dystrophy type 1	Before after 12	Yes Not specified	To determine the effect and safety of 12 weeks of aerobic training in patients with DM1.	Home based 12-week aerobic training on an ergocycle.	Improvements in VO_2_ max, maximal workload and heart rate while exercising. Greater cross-sectional area of muscle fibers. No significant changes in muscle fiber type or capillary density.
Tollbäck et al. [24]	1999 Sweden	Effects of high resistance training in patients with myotonic dystrophy.	Before after 9	Not specified Adult onset	To evaluate effects of dynamic high-resistance training program on muscle strength, muscle area and muscle fiber histopathology in ambulatory patients with DM1.	12-week supervised quadriceps strength training program.	Strength improvements in 1 RM but no significant changes in isokinetic testing. Significant increased CNF. Trend shows increased CSA of muscle fibers and a shift to type 1 muscle fibers.
Torres et al. [40]	1983 United States	Quantitative testing of handgrip strength, myotonia, and fatigue in myotonic dystrophy	Before after 10	N/A Not specified	To describe strength endurance, myotonia and fatigue in DM1 vs controls.	Acute prehension exercises.	Decreased strength, increased relaxation time and increased time to fatigue in DM1 vs controls.

Legend: n represents the number of DM1 participants included in the study. Some studies included other neuromuscular diseases or unaffected individuals; these were not presented here. Genetic testing was considered not applicable in studies before 1992 because the responsible genetic mutation had not yet been discovered before this date

^a^Phenotype reported as described in the study, no further precisions were available

### Exercise and training protocols

(Left column of Additional file 2). Different exercise or training protocols have been used to study muscle responses or adaptations. As expected, single session exercises were done under supervision [31, 34, 36–40]. Regarding training programs, seven were done under healthcare professional supervision [24, 29, 33, 43–45], two presented a mixed supervision (professionally supervised and home-based sessions) [28, 41], one was done under parent’s supervision [42] and four were performed at home, then without supervision [2, 3, 30, 32].

### Clinical measurements

(Upper row – left of Additional file 2). Many standardized functional tests were used (Additional file 2), some studies used technology to assess function (Missaoui et al. 2010 [43]: a Statel stabilometer to assess balance and a locometer as a computer assisted movement analysis to evaluate gait) and homemade clinical functional tests such as measuring the time needed to do transfers (Lindeman et al. 1995 [30] and Conraads et al. 2002 [45]). Muscle endurance testing, strength testing, aerobic capacity and other clinical measurements are further detailed in Additional file 2. Many different clinical measurements were given throughout the selected studies and most of them relied on highly standardized protocols, however, this is not always the case. Therefore, the studies that included these types of measures were listed and reproducibility of their protocol was evaluated (Table 3).

**Table 3:** Clinical measurements standardization

	Muscle endurance		Aerobic capacity		Strength			
	Time at 50 % max strength	Time at 80 % max strength	Submaximal cycling and walking	VO_2_ max (cycling), heart rate and gas exchanges	1 RM	Isokinetic and isometric computer assisted device	MMT	Force gauge (handheld or fixed)
Aldehag 2005 [41]								O
Aldehag 2013 [28]								O
Chisari 2013 [32]							O	
Conraads 2002 [45]			X		X	X		
Cudia 2016 [33]							O	
Esposito 2017 [34]	O							O
Hammarén 2015 [35]								O
Lindeman 1995 [30]		O				O		
Lindeman 1999 [2]		O				X		
Missaoui 2010 [43]						X		
Nitz 1999 [36]								X
Orngreen 2005 [3]				X				
Sjögreen 2010 [42]	O							O
Tollbäck 1999 [24]					O	O		
Torres 1983 [40]	O							O

Legend: O: sufficient data for precise reproduction of protocol. X: insufficient data for precise reproduction of protocol

### Physiological variables

(Upper row – right of Additional file 2). Surface electromyography (SMEG) was the only method used to assess muscle electric activation within the included studies [2, 32, 34, 36, 40]. Esposito et al. 2017 [34] introduced mechano myography (MMG) to evaluate the degree of muscle mechanical activation. Blood tests were used by Siciliano et al. 2001 [38] to assess exercise-induced blood lactate levels as an indicator of mitochondrial function. Blood myoglobin levels were used in another study [30] to ensure that training did not induce negative effects. MRI was used to evaluate different muscular variables: 1) quadriceps muscle volume [24, 33, 44], 2) fatty infiltration of the *tibialis anterior* [33], 3) intramuscular pH [31, 39, 44] and 4) bioenergetics, such as adenosine tri-phosphate (ATP), inorganic phosphate or phosphocreatine (PCr) [31, 39, 44]. Muscle biopsy was used to assess muscle bioenergetics (ATP and PCr) in a study conducted by Rehunen et al. 1985 [37]. It was also used by Orngreen et al. 2005 [3] to determine capillary density. Then, fiber type was assessed in two different studies [24, 37] and muscle fiber cross sectional area (CSA) was also evaluated in two papers [3, 24]. The proportion of centrally nucleated fibers (CNF) was evaluated in one study [24].

### Outcome types

Main outcome types of included studies can be found in Table 2. During the analysis of the effect of exercise or training on skeletal muscle in DM1, it appeared that the type of outcomes should be analyzed through two types of intervention, as their natures are completely different: muscular responses induced by acute exercise (single session) and muscular adaptations induced by training programs are thus presented separately.

### Single session exercise

Regardless of the assessment method used, MRI or biopsy, all three studies measuring bioenergetics showed faster PCr and ATP depletion during exercise in DM1 patients in comparison to unaffected individuals [31, 37, 39]. One of these three papers showed an increased intramuscular PCr concentration at rest compared to unaffected individuals [37]. One of these papers along with one using blood tests to evaluate blood lactate levels suggested mitochondrial defects [31, 38], but Barnes et al. 1997 [31] supported that this mechanism was not the main reason why DM1 patients experienced muscle weakness and wasting. All two studies [31, 39] evaluating intramuscular pH, reported higher pH post-exercise and a quicker return to normal levels of pH post-exercise. Strength testing, in all three studies, showed lower maximal strength [34, 36, 40] and longer relaxation time compared to healthy controls [34, 36, 40], which are consistent with muscle weakness and myotonia experienced by patients with DM1. In one of two studies evaluating muscle endurance [40], a longer time before fatigue (evaluated by calculating the time that the patient is able to maintain a 50% maximal strength contraction) was reported in DM1 individuals in comparison to unaffected ones. In contrast, the other study evaluating muscle endurance, Esposito et al. 2017 [34] reported that rhythmic 6 s contractions at 50% of maximal strength led to the same time to exhaustion between DM1 and unaffected individuals. In the two studies evaluating root mean square (RMS) of electromyography (EMG) signal (an indicator of physiological electrical activity), one found RMS increased compared to unaffected individuals [36] and continued to increase with repetitions of exercise while the other found no significant differences of RMS between DM1 and control subjects during exercise [34].

### Training programs

Positive effects of training programs on patient-reported outcomes, such as improved self-perception of occupational performance, are found in all five studies measuring this outcome [3, 28, 30, 35, 41]. Lindeman et al. 1999 [2] showed a significant increase in muscle endurance while Lindeman et al. 1995 [30] showed a trend towards increased muscle endurance. In addition, Sjögreen et al. [42] showed an increase in muscle endurance in some participants. These were the only three studies that evaluated the effect of a training program on muscle endurance. Aerobic capacity was improved in both studies that evaluated it [3, 45], with one study showing an improvement in respiratory function [45]. Improvement in functional tests was found in four studies of the ten studies that included them [32, 33, 41, 43]. All the other studies showed no change except for a decreased performance in the 10 m walk test (10mwt) that was reported by Hammarén et al. 2015 [35], in which they hypothesized it was due to a greater caution from the patients because they might be better aware of the risk of falling. Maximal strength improvement was reported in nine out of eleven studies that measured it [24, 28, 32, 33, 35, 41–43, 45]. It is to be noted that in some of these studies, strength improvement was not observed in all participants, in all muscle groups trained or in different assessment methods [24, 28, 32, 33, 35, 41, 42]. One study reported loss in maximal strength in some patients [35]. One study out of two showed an exercise-induced normalization of electromyographic patterns through SMEG evaluation [32]. Bioenergetics (ATP and PCr) were normalized without a change in abnormal pH in the only study that evaluated it following a training program [44]. One study out of three reported a slight increase in muscle volume through MRI [44]. The two studies that used muscle biopsies to assess muscle fiber CSA reported an increase, one only a trend in type I fibers [24] and one in both type I and type IIa [3]. These findings are encouraging because they support muscle growth. Fiber type and capillary density were not reported to change [3, 24]. The only study evaluating these factors reported an increase in CNF [24] and unchanged blood myoglobin levels were also [30].

## Discussion

**Fig. 2:**
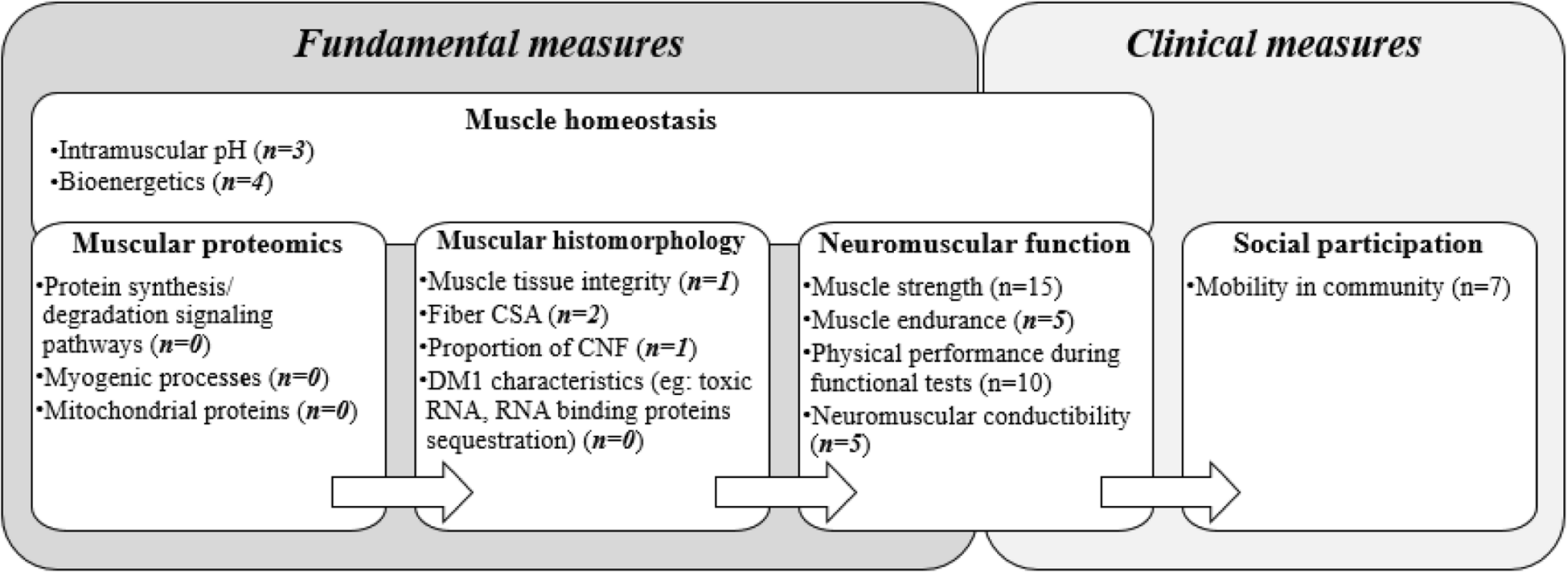
Mapping of current literature according to their type of measures. Each category (in bold) includes domains that can be influenced by exercise or training. Number of publications (n) addressing each of these domains is indicated in parentheses (in bold and italic if this number is ≤5). CSA, Cross sectional area; CNF, centrally nucleated fibers

The aim of this scoping review was to map out what is known about the effects of exercise or training program used to counteract muscle impairments in people affected by DM1 in order to guide future research. There is an obvious lack of studies available to fully comprehend muscular immediate responses and long-term adaptations to exercise or training and some domains have been insufficiently or have not yet been investigated (Fig. 2). More literature related to exercise or training could be particularly useful for healthcare professionals, such as physical therapists, who need to know the right type and dosage of exercise to prescribe to their patients to limit the progression of muscle impairments. Although the majority of studies report no adverse effects of exercise or training, there is no evidence supporting positive effects and data are insufficient to have a clear understanding of the optimal parameters to use with this population. A previous systematic review has also showed no adverse effects of strength training in people with DM1 [25].

Being a scoping review, the methodological quality of the different studies has not been assessed and this constitutes a limit to the interpretation of the outcomes reported in the different studies. Furthermore, as the exercise and training protocols and outcome measures were highly heterogenous, data pooling is not yet a feasible option. As shown in this scoping review, there is no sufficient similar high-quality evidences available to carry out a systematic review or a meta-analysis with a research question such as “What is known about the effects of exercise or training that aim to reduce skeletal muscle impairments of patients with myotonic dystrophy type 1?”. One preferred design seems to be before/after or crossover designs, which may be better suited for studies where few participants are available [46].

Exercise and training protocols have been separated into two types to facilitate the analysis. Single session exercise studies allowed the understanding of the early muscle responses to exercise of people with DM1 in comparison to controls. On the other hand, training programs gave information about muscular adaptations. These can lead to a better understanding of the benefits of long-term training and thus help healthcare professionals to choose an appropriate rehabilitation approach to manage skeletal muscle impairments. Studies using single session exercise have used more physiological variables to assess efficacy while studies using training programs tended to use more clinical measurements. This is not surprising since a short (unique) stimulus is not expected to induce clinically significant changes. However, the inclusion of physiological variables in studies using training programs would have given an important insight of the cellular and molecular mechanisms supporting the changes in clinical outcomes. This scoping review has outlined that the focus in the physiological variables was on muscle conductivity, bioenergetics, mitochondrial activity, intramuscular pH, centrally nucleated fibers and muscle fiber type and size. While it is known in DM1 that skeletal muscle has multiple alterations in inflammatory status, myogenesis and protein synthesis/degradation balance [1, 18–22], there are, to this day, no studies that consider the impact of exercise or training on these aspects. This knowledge would be a key component to help to understand why patients do not all show the same response to exercise or training throughout the different studies, thus leading to a better comprehension of the dosage and type of exercise or training that should be prescribed to obtain optimal results.

It is to be noted that at the time period when the systematic search was conducted, no animal studies about the effect of exercise in DM1 were found. This scoping review was thus directed on human studies only. It is only very recently (January 2019) that the first animal model study was published to assess the effect of exercise in DM1 (access to a running wheel for 7 weeks) by Manta et al. [47] This study demonstrates very interesting results about the effect of exercise in DM1: it improves strength and endurance, normalizes electromyographic signal (an indication of reduced myotonia), increases mitochondrial content, decreases accumulation of CUG RNA resulting in a decreased sequestration of MBNL-1 among other physiological benefits [47]. These results are consistent with some uncovered in this scoping review where exercise had beneficial effects on strength [24, 28, 32, 33, 35, 41–43, 45], endurance [2] or electromyographic signal [32]. However, many of the physiological outcomes measured by Manta et al. have not yet been measured in humans, showing another gap in current scientific literature.

From a clinical standpoint, this scoping review showed that there is a major lack in the body of evidence available to help clinicians to base their rehabilitation interventions on solid literature for their DM1 patients. In addition, compliance of patients to participate to a rehabilitation intervention therapy can be hampered by other symptoms since DM1 is a multisystemic disease [9]. For example, it has been shown that 40% of patients with DM1 suffer from apathy [48]. This can have a major impact on the reported outcome of exercise and training. Cardiac impairments are also frequently reported in DM1 [49], which can lead to restrictions in exercise and training participation. It is worth to be mentioned that these are only a few examples of other symptoms of DM1 that can interfere with exercise or training, which should be investigated in the future.

A lack in the standardization of some clinical measurements has also been observed between the selected studies. This element is particularly important to control with DM1 patients since many other confounding factors arising from the multisystemic nature of the disease may already influence the results. An example of this is strength testing, which represents one of the major outcomes used to assess muscle adaptations following a training program. The issue about lower limb strength assessment in DM1 patients has already been evaluated in further detail by a systematic review [50] where different methods were described and very few of them had sufficient methodological descriptions to allow protocol reproducibility. In addition, manual muscle testing has been shown to significantly lack sensibility and validity to adequately measure muscle strength in DM1 population [8]. In this scoping review, only two studies out of 15 that evaluate maximal strength used only MMT to assess strength; however, this still represents 9% of the accepted studies. Furthermore, these two studies were the only ones using a NMES training program, which greatly hampers the strength of the evidence regarding this type of training.

Another interesting point brought on by Petitclerc et al. 2017 is that adult and late-onset phenotypes should not be pooled together to assess muscle strength because of their different maximal strength loss profile over time [8]. Congenital, infantile, childhood onset phenotypes are already known to be even more clinically different from the adult and late-onset phenotypes [49]. This brings the hypothesis that some methodological risks are associated to the pooling of different phenotypes when evaluating the effectiveness of an intervention aimed at decreasing muscular impairments. In this scoping review, many studies mixed different phenotypes or do not even specify the phenotypes of their participants. Future studies should consider this important element when recruiting their participants. Moreover, many studies published after 1992 did not specify nor did genetic testing to validate if their participants were true DM1 patients. As the mechanisms underlying maximal muscle strength loss vary greatly between neuromuscular diseases, genetic testing should be a standard procedure in DM1 research.

## Conclusion

This paper maps and summarizes all existent literature about the effect of exercise or training in humans with DM1. We also offer a clear framework to guide future studies. There is a great deal of research that still needs to be done in order to fully understand how skeletal muscles in DM1 patients respond to exercise and training. Many studies looked at neuromuscular function, however there are still an insufficient number of RCTs to come to clear conclusions. Furthermore, no studies have yet evaluated the effects of exercise or training on muscle proteomics, that are essential to understand the underlying mechanisms of the observed adaptations. Once a better understanding will be reached, clinicians will be able to prescribe interventions more effectively and thus better manage muscle wasting and weakness.

## Additional file

Additional file 1: Search words and headings. All search words and headings used in the different data bases (Medline (EBSCO), Pubmed, CINAHL (EBSCO) and EMBASE) can be found in this additional file. (DOCX 19 kb)

Additional file 2: Summary of measurements, parameters and exercise or training protocols. (PDF 162 kb)

## Abbreviations

10mwt: 10-m walk test
1RM: One maximal repetition test
6mwt: 6-min walk test
ATP: Adenosine triphosphate
BBS: Berg balance scale
CNF: Centrally nucleated fibers
CoQ10: Coenzyme Q10
CSA: Cross sectional area
CTG: Cytosine, thymine and guanine nucleotide triplet
DM1: Myotonic dystrophy type 1
*DMPK*: Dystrophy Myotonic Protein Kinase gene
EMG: Electromyography
GSK3β: Glycogen synthase kinase 3 beta
MMG: Mechanomyography
MMT: Manual muscle testing
MRI: Magnetic resonance imaging
NMES: Neuromuscular electrical stimulation
PCr: Phosphocreatine
RCT: Randomized controlled trial
RNA: Ribonucleic acid
SMEG: Surface electromyography
TUG: Timed up and go
VO_2_ max: Maximal volume of oxygen
