# Correction to: What is known about the effects of exercise or training to reduce skeletal muscle impairments of patients with myotonic dystrophy type 1? A scoping review

**DOI:** 10.1186/s12891-019-2643-8

**Published:** 2019-05-24

**Authors:** Marie-Pier Roussel, Marika Morin, Cynthia Gagnon, Elise Duchesne

**Affiliations:** 1 Département des sciences de la santé, physiothérapie, Université du Québec à Chicoutimi, 555, boulevard de l’Université, Chicoutimi, Quebec G7H 2B1 Canada; 2 Groupe de recherche interdisciplinaire sur les maladies neuromusculaires, Centre intégré universitaire de santé et de services sociaux du Saguenay–Lac-St-Jean, 2230 rue de l’Hôpital, Saguenay, Québec Canada; 3 Centre de recherche Charles-Le Moyne – Saguenay–Lac-Saint-Jean sur les innovations en santé, 2230 rue de l’Hôpital, Saguenay, Québec, Canada, Longueuil, Québec Canada; 4 Faculté de médecine et des sciences de la santé, Université de Sherbrooke, 3001, 12e Avenue Nord, Sherbrooke, Québec Canada

**Correction to: BMC Musculoskelet Disord (2019) 20:101**

**https://doi.org/10.1186/s12891-019-2458-7**

## Abstract

An incorrect attribution of the first study regarding the effect of exercise in DM1 mouse models needs to be revised.

## Main text

In this article, we cited Manta et al. 2019 [1] as the first study to publish about the effects of exercise in DM1 mouse models This was an oversight considering that Ravel-Chapuis et al. 2018 [2] have reported in the last part of their “Results” section, presentation of the effects of wheel running on skeletal muscles of their DM1 mouse model. This article was published in October 2018 while the article of Manta et al. 2019 [1] was published in January 2019.
