# Supplementary material for: What is known about the effects of exercise or training to reduce skeletal muscle impairments of patients with myotonic dystrophy type 1? A scoping review
Source: BMC Musculoskelet Disord. 2019 Mar 5;20:101. doi: 10.1186/s12891-019-2458-7 (PMC6402179; doi:10.1186/s12891-019-2458-7)
Supplement: Supplementary file 1 — Search words and headings. All search words and headings used in the different data bases (Medline (EBSCO), Pubmed, CINAHL (EBSCO) and EMBASE) can be found in this additional file. (DOCX 19 kb) [file 12891_2019_2458_MOESM1_ESM.docx]

## Additional file 1- Search words and headings

|  |  | Concept 1 | Concept 2a | Concept 2b | Concept 3 |
| --- | --- | --- | --- | --- | --- |
| Medline via Pubmed | Headings | Myotonic Dystrophy | Physical fitness  Exercise therapy  Exercise  Resistance training | Rehabilitation  Occupational therapy  Physical therapy modalities | Muscles with subheading   - Anatomy and histology - Etiology - Physiopathology   Muscle development with subheading   - Etiology - Physiology   Muscular atrophy |
|  | Synonyms | DM1  Dystrophia myotonica  Steinert's disease  Steinert disease  Myotonic dystrophy | Exercise therapy  Exercise therapies  Physical fitness  Physical Conditioning  Conditioning human  Exercise  Exercises  Physical exercise  Physical exercises  Strength training | Therapy  Therapies  Rehabilitation  Habilitation  Occupational therapy  Physical therapy  Physiotherapy  Occupational therapies  Physical therapies  Physiotherapies | Muscle  Muscles  Muscle impairment  Muscle impairments  Muscular Impairments  Muscular Impairments  Muscle histology  Muscular histology  Myogenesis  Muscle development  Muscular Development  Myofibrillogenesis  Muscle atrophy  Muscular atrophy  Muscle atrophies  Muscular atrophies |
| Medline via EBSCO | Headings | Myotonic Dystrophy | Physical fitness  Exercise therapy  Resistance training | Rehabilitation  Occupational therapy  Physical therapy modalities | Muscles with subheading   - Abnormalities - Anatomy and histology - Immunology - Pathology - Physiology - Physiopathology   muscle development with subheading   - Genetics - Immunology - Physiology   muscular atrophy |
|  | Synonyms | DM1  Dystrophia myotonica  Steinert's disease  Steinert disease  Myotonic dystrophy | Exercise therapy  Exercise therapies  Physical fitness  Physical Conditioning  Conditioning human  Exercise  Exercises  Physical exercise  Physical exercises  Strength training | Therapy  Therapies  Rehabilitation  Habilitation  Occupational therapy  Physical therapy  Physiotherapy  Occupational therapies  Physical therapies  Physiotherapies | Muscle  Muscles  Muscle impairment  Muscle impairments  Muscular Impairments  Muscular Impairments  Muscle histology  Muscular histology  Myogenesis  Muscle development  Muscular Development  Myofibrillogenesis  Muscle atrophy  Muscular atrophy  Muscle atrophies  Muscular atrophies |
| CINAHL via EBSCO | Headings | Myotonic dystrophy | [Muscle Strengthening](javascript:XslPostBack('ctl00$ctl00$MainContentArea$MainContentArea$ctrlResults','meshDetail','index%7C3%24term%7CMuscle%20Strengthening%24cmd%7CmeshDetail');)  [Resistance training](javascript:XslPostBack('ctl00$ctl00$MainContentArea$MainContentArea$ctrlResults','meshList','index%7C4%24term%7CResistance%20Training%24cmd%7CmeshList');)  [Physical fitness](javascript:XslPostBack('ctl00$ctl00$MainContentArea$MainContentArea$ctrlResults','meshDetail','index%7C1%24term%7CPhysical%20Fitness%24cmd%7CmeshDetail');)  [Physical activity](javascript:XslPostBack('ctl00$ctl00$MainContentArea$MainContentArea$ctrlResults','meshDetail','index%7C5%24term%7CPhysical%20Activity%24cmd%7CmeshDetail');)  [Exercise](javascript:XslPostBack('ctl00$ctl00$MainContentArea$MainContentArea$ctrlResults','meshDetail','index%7C6%24term%7CExercise%24cmd%7CmeshDetail');) | [Physical therapy](https://web-a-ebscohost-com.acces.bibl.ulaval.ca/ehost/mesh/tree?term=Physical%20Therapy&sid=4bc18622-b054-4b19-97d0-e81b0212f304%40sessionmgr4002&vid=10)  [Rehabilitation](https://web-a-ebscohost-com.acces.bibl.ulaval.ca/ehost/mesh/tree?term=Rehabilitation&sid=4bc18622-b054-4b19-97d0-e81b0212f304%40sessionmgr4002&vid=10)  [Occupational Therapy](javascript:XslPostBack('ctl00$ctl00$MainContentArea$MainContentArea$ctrlResults','meshDetail','index%7C1%24term%7COccupational%20Therapy%24cmd%7CmeshDetail');) | [Muscle, skeletal](https://web-b-ebscohost-com.acces.bibl.ulaval.ca/ehost/mesh/tree?term=Muscle%2C%20Skeletal&sid=467e0e2a-bb5f-4185-91a6-69a61cdc30b5%40sessionmgr115&vid=194) with subheading   - Abnormalities - Analysis - Anatomy and histology - Immunology - Pathology - Physiology - Physiopathology   [Muscular atrophy](https://web-b-ebscohost-com.acces.bibl.ulaval.ca/ehost/mesh/tree?term=Muscular%20Atrophy&sid=467e0e2a-bb5f-4185-91a6-69a61cdc30b5%40sessionmgr115&vid=197)  [Muscle weakness](javascript:XslPostBack('ctl00$ctl00$MainContentArea$MainContentArea$ctrlResults','meshDetail','index%7C11%24term%7CMuscle%20Weakness%24cmd%7CmeshDetail');)  [Exercise physiology](https://web-b-ebscohost-com.acces.bibl.ulaval.ca/ehost/mesh/tree?term=Exercise%20Physiology&sid=467e0e2a-bb5f-4185-91a6-69a61cdc30b5%40sessionmgr115&vid=223) |
|  | Synonyms | DM1  Dystrophia myotonica  Steinert's disease  Steinert disease  Myotonic dystrophy | Exercise therapy  Exercise therapies  Physical fitness  Physical Conditioning  Conditioning human  Exercise  Exercises  Physical exercise  Physical exercises  Strength training | Therapy  Therapies  Rehabilitation  Habilitation  Occupational therapy  Physical therapy  Physiotherapy  Occupational therapies  Physical therapies  Physiotherapies | Muscle  Muscles  Muscle impairment  Muscle impairments  Muscular Impairment  Muscular Impairments  Muscle histology  Muscular histology  Myogenesis  Muscle development  Muscular Development  Myofibrillogenesis  Muscle atrophy  Muscular atrophy  Muscle atrophies  Muscular atrophies |
| EMBASE | Headings | [Myotonic dystrophy](http://www.embase.com.acces.bibl.ulaval.ca/) | [Training](http://www.embase.com.acces.bibl.ulaval.ca/)  [Muscle training](http://www.embase.com.acces.bibl.ulaval.ca/)  [Resistance training](http://www.embase.com.acces.bibl.ulaval.ca/)  [Isometric exercise](http://www.embase.com.acces.bibl.ulaval.ca/) | [Physiotherapy](http://www.embase.com.acces.bibl.ulaval.ca/)  [Occupational therapy](http://www.embase.com.acces.bibl.ulaval.ca/)  [Rehabilitation](http://www.embase.com.acces.bibl.ulaval.ca/) | [Muscle atrophy](http://www.embase.com.acces.bibl.ulaval.ca/)  [Muscle weakness](http://www.embase.com.acces.bibl.ulaval.ca/)  [Skeletal muscle](http://www.embase.com.acces.bibl.ulaval.ca/)  [Muscle biopsy](http://www.embase.com.acces.bibl.ulaval.ca/)  [Muskuloskeletal](http://www.embase.com.acces.bibl.ulaval.ca/emtree)  [Musculoskeletal](http://www.embase.com.acces.bibl.ulaval.ca/emtree)  [Muscle development](http://www.embase.com.acces.bibl.ulaval.ca/)  [Histology](http://www.embase.com.acces.bibl.ulaval.ca/) |
|  | Synonyms | DM1  Dystrophia myotonica  Steinert's disease  Steinert disease  Myotonic dystrophy | Exercise therapy  Exercise therapies  Physical Fitness  Physical Conditioning  Conditioning human  Exercise  Exercises  Physical exercise  Physical exercises  Strength training | Therapy  Therapies  Rehabilitation  Habilitation  Occupational therapy  Physical therapy  Physiotherapy  Occupational therapies  Physical therapies  Physiotherapies | Muscle  Muscles  Muscle impairment  Muscle impairments  Muscular Impairment  Muscular Impairments  Muscle histology  Muscular histology  Myogenesis  Muscle development  Muscular Development  Myofibrillogenesis  Muscle atrophy  Muscular atrophy  Muscle atrophies  Muscular atrophies |
