# Supplementary material for: What is known about the effects of exercise or training to reduce skeletal muscle impairments of patients with myotonic dystrophy type 1? A scoping review
Source: BMC Musculoskelet Disord. 2019 Mar 5;20:101. doi: 10.1186/s12891-019-2458-7 (PMC6402179; doi:10.1186/s12891-019-2458-7)
Supplement: Supplementary file 2 — Summary of measurements, parameters and exercise or training protocols. (PDF 162 kb) [file 12891_2019_2458_MOESM2_ESM.pdf]

Table 2: Summary of measurements, variables and exercise or training protocols.

Outcome measures: Clinical measurements

Exercise or training protocols		Patient-reported outcome measures	Homemade	Validated	Functional tests	6-minute walk test (6mwt)	10 m walk test (10mwt)	Berg balance scale (BBS)	Computer assisted movement analysis	Perdue pegboard test	SVANTE articulation test	Homemade functional tests	Step test	Time up and go (TUG)	Time stands test (TST)	Muscle endurance	Time at 50 % max strength	Time at 80 % max strength	Aerobic capacity	Submaximal cycling and walking	VO_2 max (cycling), heart rate and gas exchanges	Muscle strength	1 RM	Isokinetic/isometric computer assisted device	MMT	Force gauge (handheld or fixed)	Other	Body mass index	MIRS	Respiratory function
Single session exercise	Barnes 1997[28]																													
Single session exercise	Esposito 2017[31]																x									x				
Single session exercise	Nitz 1999[33]																									x				
Single session exercise	Rehunen 1985[34]																													
Single session exercise	Siciliano 2001[35]																													
Single session exercise	Taylor 1993[36]																													
Single session exercise	Torres 1983[38]																x									x				
Training programs	**NMES**																													
Training programs	Chisari 2013[29]					x	x							x											x					
Training programs	Cudia 2016[30]					x	x																		x				x	
Training programs	**Strength training**																													
Training programs	Aldehag 2005[39]		x	x						x																x				
Training programs	Aldehag 2013[25]		x	x						x																x			x	
Training programs	Lindeman 1995[27]		x			x						x						x						x						
Training programs	Lindeman 1999[2]																	x						x						
Training programs	Sjögreen 2010[40]		x						x		x						x									x				
Training programs	Tollbäck 1999[37]																						x	x						
Training programs	**Aerobic training**																													
Training programs	Orngreen 2005[3]		x																		x									
Training programs	Trenell 2006[42]																													
Training programs	**Balance training**																													
Training programs	Hammarén 2015[32]		x	x			x						x	x												x				
Training programs	**Multiple rehabilitation interventions**																													
Training programs	Conraads 2002[43]											x								x			x	x						x
Training programs	Kierkegaard 2011[26]		x	x		x								x	x													x		
Training programs	Missaoui 2010[41]		x					x	x					x										x						
	**Total**		8	4		4	3	1	2	2	1	2	1	4	1		3	2		1	1		2	5	2	7		1	2	1

Outcome measures: Physiological variables

Exercise or training protocols		Myography	Muscle mechanical activation (MMG)	Muscle electric activation (SMEG)	MRI	Bioenergetics	Fat infiltration	Muscle volume	pH	Muscle biopsy	Bioenergetics	Capillary density	Centrally nucleated fibers (CNF)	Fiber CSA	Fiber type	Blood tests	Lactate	Myoglobin
Single session exercise	Barnes 1997[28]					x			x									
Single session exercise	Esposito 2017[31]		x	x														
Single session exercise	Nitz 1999[33]			x														
Single session exercise	Rehunen 1985[34]										x				x			
Single session exercise	Siciliano 2001[35]																x	
Single session exercise	Taylor 1993[36]					x			x									
Single session exercise	Torres 1983[38]			x														
Training programs	**NMES**																	
Training programs	Chisari 2013[29]			x														
Training programs	Cudia 2016[30]						x	x										
Training programs	**Strength training**																	
Training programs	Aldehag 2005[39]																	
Training programs	Aldehag 2013[25]																	
Training programs	Lindeman 1995[27]																	x
Training programs	Lindeman 1999[2]			x														
Training programs	Sjögreen 2010[40]																	
Training programs	Tollbäck 1999[37]							x					x	x	x			
Training programs	**Aerobic training**																	
Training programs	Orngreen 2005[3]											x		x				
Training programs	Trenell 2006[42]					x		x	x									
Training programs	**Balance training**																	
Training programs	Hammarén 2015[32]																	
Training programs	**Multiple rehabilitation interventions**																	
Training programs	Conraads 2002[43]																	
Training programs	Kierkegaard 2011[26]																	
Training programs	Missaoui 2010[41]																	
	**Total**		1	5		3	1	3	3		1	1	1	2	2		1	1
